# Resistin and interleukin-6 exhibit racially-disparate expression in breast cancer patients, display molecular association and promote growth and aggressiveness of tumor cells through STAT3 activation

**DOI:** 10.18632/oncotarget.3591

**Published:** 2015-03-14

**Authors:** Sachin K. Deshmukh, Sanjeev K. Srivastava, Arun Bhardwaj, Ajay P. Singh, Nikhil Tyagi, Saravanakumar Marimuthu, Donna L. Dyess, Valeria Dal Zotto, James E. Carter, Seema Singh

**Affiliations:** ^1^ Department of Oncologic Sciences, Mitchell Cancer Institute, University of South Alabama, Mobile, Alabama, USA; ^2^ Department of Biochemistry and Molecular Biology, College of Medicine, University of South Alabama, Mobile, Alabama, USA; ^3^ Department of Pathology, College of Medicine, University of South Alabama, Mobile, Alabama, USA

**Keywords:** racial disparity, breast cancer, resistin, IL-6, STAT3, inflammatory cytokine

## Abstract

African-American (AA) women with breast cancer (BC) are diagnosed with more aggressive disease, have higher risk of recurrence and poorer prognosis as compared to Caucasian American (CA) women. Therefore, it is imperative to define the factors associated with such disparities to reduce the unequal burden of cancer. Emerging data suggest that inherent differences exist in the tumor microenvironment of AA and CA BC patients, however, its molecular bases and functional impact have remained poorly understood. Here, we conducted cytokine profiling in serum samples from AA and CA BC patients and identified resistin and IL-6 to be the most differentially-expressed cytokines with relative greater expression in AA patients. Resistin and IL-6 exhibited positive correlation in serum levels and treatment of BC cells with resistin led to enhanced production of IL-6. Moreover, resistin also enhanced the expression and phosphorylation of STAT3, and treatment of BC cells with IL-6-neutralizing antibody prior to resistin stimulation abolished STAT3 phosphorylation. In addition, resistin promoted growth and aggressiveness of BC cells, and these effects were mediated through STAT3 activation. Together, these findings suggest a crucial role of resistin, IL-6 and STAT3 in BC racial disparity.

## INTRODUCTION

Breast cancer (BC) remains the most frequently diagnosed cancer and second leading cause of cancer death in women in the United States [1]. According to the American Cancer Society, approximately 231,840 women will be diagnosed with BC and nearly 40,290 will die with this malignancy in 2015 [1]. Epidemiological data also suggest that women of African American (AA) background are disproportionately affected by BC relative to the Caucasian American (CA) women [2-4]. AA women have early onset of BC, are diagnosed with more aggressive and metastatic disease, respond poorly to therapy, have higher risk of recurrence and worse prognosis as compared to CA patients [4]. Despite these recognitions, we still do not have a clear understanding of the biological causes and associated molecular mechanisms involved in such disparity.

It is being increasingly appreciated that molecular differences at the genetic level (gene mutations, deletions, etc.) may not alone be responsible for the observed disparity in breast and other cancers, but tumor microenvironment (TME) may also play an important role in the overall outcome [5]. In this regard, efforts are focused on identifying the intrinsic differences in TME and characterizing underlying biological and molecular regulatory factors. For example, obesity and inflammation have been associated with possible ethnic/racial differences in BC survival and data continue to emerge to further support this notion [3, 6]. As per a report from Center for Disease Control and Prevention (CDC), AA population has 51 % higher obesity rates compared with CA [7]. Obesity is associated with a low-grade chronic inflammation, characterized by increased circulating fatty acids and chemo-attraction of immune cells [8]. This inflammatory microenvironment is believed to support the growth of tumor cells, promote their aggressiveness and alter their therapeutic responses. In the same line, a recent study identified distinct patterns in TME (vascularization and macrophage infiltration) of AA and CA BC patients [9] further supporting its role in BC racial disparity.

TME contains a variety of non-tumor cells including endothelial cells, fibroblasts, adipocytes and immune cells (resident and infiltrated) that cooperate in tumor development and progression by producing growth factors and cytokines [10, 11]. Resistin, originally described as an adipocyte-derived cytokine, is mostly expressed by the macrophages in humans [12, 13]. It has potent pro-inflamatory properties and considered as a potential mediator in obesity-associated diseases, including cancer [14-16]. In a recent comprehensive differential gene-expression analysis, resistin transcript was identified to be expressed at greater level in AA BC as compared to CA BC patients [17]. Similarly, IL-6 is also an inflammation-associated pleiotropic cytokine, which can be secreted by a wide array of immune, endothelial as well as cancer cells in an inducible manner [18]. IL-6-KO mice studies suggest that it plays an essential role in peripheral T-cell development, T-cell activity and lymphocyte differentiation [19]. STAT3 (signal transducer and activator of transcription 3) is a transcription factor activated in many malignancies, including BC [20-22]. Importantly, IL-6 is an important inducer of STAT3 that supports the survival of cells under an inflammatory environment [23].

In the present study, we have conducted cytokine profiling of serum samples from patients of AA or CA racial background and identified resistin and IL-6 to be most differentially-expressed cytokines exhibiting greater expression in AA patients. We also show that the treatment of BC cells with resistin leads to enhanced expression and phosphorylation of STAT3 as well as promotes IL-6 production. We further show that IL-6 mediates resistin-induced phosphorylation of STAT3. In additional findings, we demonstrate that resistin promotes growth and aggressiveness of BC cells, and these effects are mediated through STAT3 induction. These are important findings and support a cooperative role of cytokines-transcription factor network in BC racial disparity.

## RESULTS

### AA breast cancer patients exhibit significantly higher serum levels of resistin and IL-6 as compared to CA patients

**Figure 1:**
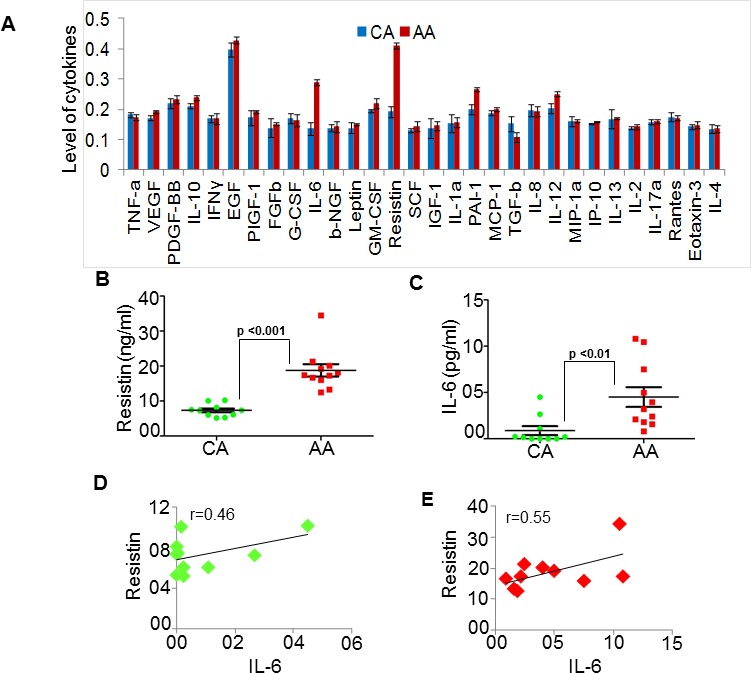
Resistin and IL-6 levels are high in serum of African American (AA) as compared to Caucasian American (CA) breast cancer patients (A) Expression profile of various cytokines was analyzed by ELISA in pooled serum samples from BC patient with CA (n=5) and AA (n=5) racial background. Levels of cytokine were plotted against the respective absorbance measured at 450 nm. (B-C) Subsequently, in a pilot study, we measured the levels of (B) resistin and (C) IL-6 in individual serum samples from CA (n=10) and AA (n=11) patients with BC. Absorbance was measured at 450 nm and amount of resistin and IL-6 was calculated using respective standards. (D-E) Correlation between resistin and IL-6 level in (D) CA and (E) AA serum samples was calculated using Pearson correlation analysis. Data indicate that resistin as well as IL-6 levels are high in serum of AA BC patients as compared to CA (p<0.01) and greater correlation between resistin and IL-6 in AA BC patients.

Considering the emerging notion that TME may play an important role in BC racial disparity, we performed cytokine profiling in pooled serum samples from five BC patients of AA or CA racial background by ELISA. We observed that resistin and IL-6 were the most differentially-expressed cytokines in serum of AA and CA BC patients with significantly greater expression in AA BC patients (Fig. 1A). To further validate these findings, we measured resistin and IL-6 levels in individual serum samples from AA (n=11) and CA (n=10) BC patients. The data demonstrate that AA BC patients have significantly higher levels of resistin (18.788 ± 1.76 ng/ml) as well as IL-6 (4.51 ± 1.07 pg/ml) as compared to that in their CA counterparts (7.33 ± 0.56 ng/ml and 0.88 ± 0.48 pg/ml, respectively) (Fig. 1B and 1C). We next subjected the expression data to Pearson correlation coefficient analysis. Our data demonstrate that the levels of resistin and IL-6 exhibit a positive correlation in serum samples, which is relatively higher in AA (r=0.55) as compared to that in CA (r=0.46) BC patients (Fig. 1D and 1E). These findings suggest a clinical association of resistin and IL-6 with BC racial disparity.

### Resistin induces IL-6 expression in breast cancer cells

**Figure 2:**
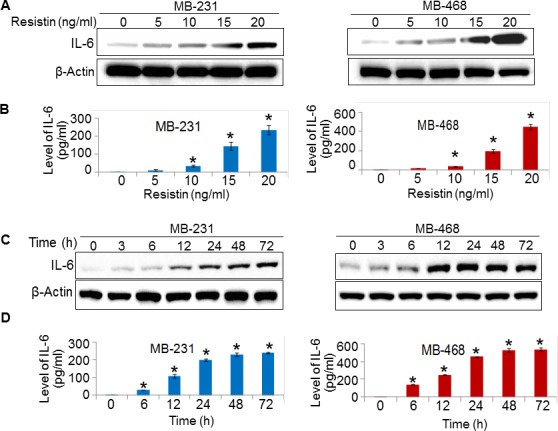
Resistin treatment enhances the expression of IL-6 (A-D) MDA-MB-231 and MDA-MB-468 cells were grown in six-well plate and treated with various doses of resistin (0-20 ng/ml) for 48 h (A, B) or with a constant dose of resistin (20 ng/ml) for indicated time intervals (C, D). Subsequently, cultured supernatants were collected and cells were lysed for protein extraction. Expression of IL-6 was examined by immunoblot assay in protein lysates (A, C) and by ELISA in culture supernatant (B, D). β-actin was used as internal control. Bars (mean ± SD, n=3) represent the level of IL-6 (pg/ml) in culture supernatant of vehicle or resistin treated BC cells. *p<0.05. IL-6 expression increased in a dose- and time- dependent manner upon resistin treatment.

Having observed a positive correlation between resistin and IL-6 in clinical cases, we examined if resistin has any role in IL-6 regulation or vice versa. For this, we first studied the expression of resistin, IL-6, and their receptors (CAP1 and IL-6R, respectively) in a panel of established BC cell lines of AA and CA origin. The data show that all the breast cancer cell lines express varying levels of IL-6, CAP1 and IL-6R (Supplementary Fig. 1), whereas no expression of resistin is observed either at the protein or transcript level in any of the cell lines (data not shown). Interestingly, higher expression of CAP1, IL-6 and IL-6R were observed in AA BC cell lines comparing to that in CA BC cell lines (Supplementary Fig. 1). We next treated two breast cancer cell lines, MDA-MB-231 (CA origin) and MDA-MB-468 (AA origin) with different doses (0-20 ng/ml) of recombinant human resistin (rh-resistin). Our data demonstrate that upon resistin treatment IL-6 was upregulated in a dose-dependent manner as observed in immunoblot (Fig. 2A) and ELISA (Fig. 2B). We next performed a time-course study by treating the breast cancer cells with rh-resistin for various time intervals. The data demonstrate that slight upregulation of IL-6 occurs as early as 3 h which continues to rise till 72 h (Fig. 2C and 2D). Interestingly, a greater induction of IL-6 was observed in MDA-MB-468 cells in response to resistin treatment as compared to the MDA-MB-231 cells. In a separate set of experiments, we treated BC cell lines with varying doses (0-100 ng/ml) of recombinant human IL-6 (rh-IL-6) to examine its effect on resistin expression. However, we did not observe any expression of resistin in rh-IL-6-treated BC cells either at protein or transcript level (data not shown). Together, these findings suggest that resistin positively regulates the expression of IL-6 in a dose- and time-dependent manner in BC cells.

### Resistin enhances STAT3 expression and phosphorylation in breast cancer cells

**Figure 3:**
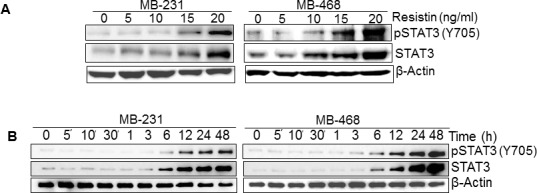
Resistin induces expression and phosphorylation of STAT3 (A-B) MDA-MB-231 and MDA-MB-468 cells were grown in 6 well plate and treated with various doses (0-20 ng/ml) of resistin at 60-70 % confluence for 48 h (A) and with a single dose of resistin (20 ng/ml) for varying time intervals (0-48 h) (B). Total protein was extracted and expression of STAT3 and pSTAT3 was examined by immunoblot assay using 40 μg total protein. β-actin was used as an internal control. Data indicate that resistin induces the expression of STAT3 as well as promotes its phosphorylation in a dose- and time- dependent manner.

STAT3 is an oncogenic transcription factor, which is overexpressed in breast and several other malignancies [20, 24, 25]. Since resistin-inducible IL-6 is a known activator of STAT3 [26], we sought out to examine the effect of rh-resistin on STAT3. MDA-MB-231 and MDA-MB-468 cells were treated with various doses of rh-resistin for a period of 48 h and its effect on STAT3 phosphorylation was examined by immunoblot analysis. The data demonstrate that STAT3 phosphorylation increases in a dose-dependent manner upon rh-resistin treatment. Interestingly, we observe that along with activation of STAT3, the expression of STAT3 is also increased in rh-resistin-treated BC cells (Fig. 3A). Next, we treated the BC cells with rh-resistin (20 ng/ml) for various time intervals. Our data show an increase in pSTAT3 and STAT3 levels after rh-resistin treatment in a time-dependent manner. The increase in expression of STAT3 and pSTAT3 is prominent from 6 h and continues to rise till 48 h (Fig. 3B). Interestingly, we observe that the extent of STAT3 and pSTAT3 induction in MDA-MB-468 cells is greater than in MDA-MB-231 cells.

### IL-6 mediates resistin-induced phosphorylation of STAT3

**Figure 4:**
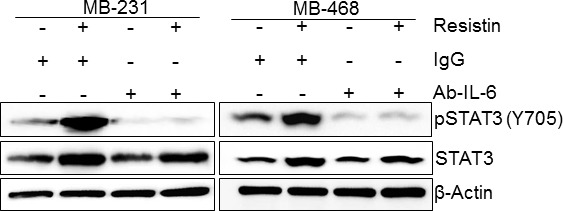
Resistin-induced phosphorylation of STAT3, but not its expression, is mediated through IL-6 Cells were treated with resistin (20 ng/ml) in presence of control IgG or IL-6 neutralizing antibody (2 μg/ml). After 48 h of treatment, total protein was isolated and expression of pSTAT3 and STAT3 was analyzed by immunoblot assay. β-actin was used as an internal control. Data show that the treatment with IL-6 neutralizing antibody abolishes basal as well as resistin-induced STAT3 phosphorylation in BC cells.

In next set of experiment, we sought out to examine if resistin-induced STAT3 expression and phosphorylation is mediated through IL-6. To test this, we treated BC cells (MDA-MB-231 and MDA-MB-468) with human IL-6 neutralizing antibody (rabbit polyclonal) prior to resistin stimulation and followed changes in STAT3 phosphorylation and expression by immunoblot assay. We also pre-treated the BC cells with rabbit IgG to serve as control. As expected, we observed induction of both STAT3 and pSTAT3 in rabbit IgG-pre-treated cells upon treatment with rh-resistin. We also observed that rh-resistin-induced phosphorylation of STAT3 was abolished in BC cells pre-treated with IL-6 neutralizing antibody, whereas only minor effect was seen on total STAT3 expression (Fig. 4). These data suggest that IL-6 primarily mediates the effects of resistin on STAT3 activation. To further confirm this, we treated the BC cells with rh-IL-6 and examined its effect on STAT3 expression and phosphorylation for various time intervals. Consistent with previous findings [27], the data reveal a quick activation (within 5 minutes) of STAT3 upon rh-IL-6 treatment, while negligible effect is observed on total STAT3 expression even at late (up to 48 h) time points (Supplementary Fig. 2).

### Resistin promotes growth and aggressive phenotype of breast cancer cells through STAT3 activation

**Figure 5:**
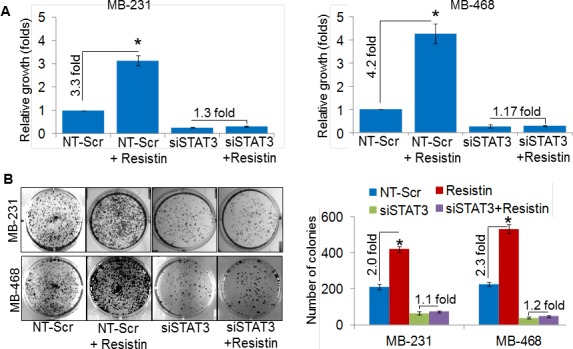
STAT3 mediates resistin-induced promotion of breast cancer cell growth and clonogenicity (A) MDA-MB-231 and MDA-MB-468 BC cells were grown in 96-well plate and transfected with NT-Scr or STAT3 targeting siRNAs. After 24 h of transfection, cells were treated with resistin (20 ng/ml) for 96 h and growth was monitored by WST-1 assay. (B) BC cells were seeded in regular media and transfected with NT-Scr or siSTAT3. After 24 h of transfection, cells were seeded at low density (1×10^3^ cells per well) in 6-well plates and treated with resistin (20 ng/ml). Culture media was replaced with fresh treatment media every 3^rd^ day. After two weeks of treatment, colonies were stained with crystal violet, visualized, photographed and counted using imaging system. Data represents mean ± SD. n=3, *, p< 0.05.

Having observed an overexpression of resistin in BC patients and its association with STAT3 upregulation, we next examined its effect on the growth and malignant phenotype of BC cells and whether these effects are mediated through STAT3. For this, we first transiently transfected BC cells with STAT3-specific (siSTAT3)- or non-targeted scrambled (NT-Scr) siRNAs for 24 h (in case of growth and clonogenicity assay) and 48 h (in case of motility and invasion assays) prior to rh-resistin treatment. STAT3 siRNAs exhibited efficient silencing efficacy within 48 h that sustained at least up to 96 h post-transfection (Supplementary Fig. 3). Resistin treatment increased the growth of MDA-MB-231 (~3.3 fold) and MDA-MB-468 (~4.2 fold) cells, whereas STAT3 silencing caused significant decrease in basal as well as rh-resistin induced growth in both the cell lines (Fig. 5A). In parallel, to monitor the effect of rh-resistin on BC cell growth in long term, we performed plating efficiency assay. Significantly enhanced plating efficiency was observed in rh-resistin treated MDA-MB-231 (~2.0 fold) and MDA-MB-468 (~2.3 folds) cells as compared to untreated cells (Fig. 5B). Furthermore, STAT3 silencing decreased the plating efficiency of BC cells, an effect, which remained unaltered following rh-resistin treatment (Fig. 5B).

**Figure 6:**
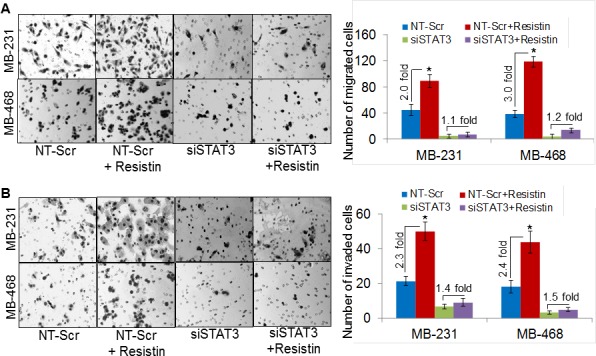
Silencing of STAT3 diminishes resistin-induced aggressiveness of breast cancer cells MDA-MB-231 and MDA-MB-468 cells were transfected with NT-Scr or STAT3-targeting siRNAs for 48 h, post transfection, cells were treated with resistin (20 ng/ml) for next 48 h and effect on cell motility (A) and invasion (B) was analyzed. (A) Cells (2.5×10^5^/well, for migration) were seeded on the top chamber of uncoated polyethylene teraphthalate or (B) for invasion assay, cells (5×10^4^/well) were seeded on top of Matrigel-coated polycarbonate membranes. Regular media containing 10 % FBS was used as a chemoattractant in lower chamber. After 16 h, migrated/invaded cells were fixed, stained and counted in 10 random view fields. Bars represent the mean ± S.D (n=3); *, p<0.05. Data show that resistin induced BC cells aggressiveness is mediated through STAT3.

Next, we investigated the role of resistin in promoting the malignant behavior of BC cells. For this, we studied the effect of resistin on motility and invasiveness, which are two important characteristics of the aggressive cancer cells [28]. Significant increase in the number of migrated cells was observed in the rh-resistin-treated MDA-MB-231 (~2.0 fold) and MDA-MB-468 (~3.0 fold) cells as compared to their respective controls (Fig. 6A). To elucidate the effect of rh-resistin on the invasive properties of BC cells, we performed *in vitro* Matrigel invasion assay. Our data demonstrate increased invasiveness of the MDA-MB-231 (~2.3-fold), and MDA-MB-468 (~2.4 fold) BC cells treated with rh-resistin as compared with control cells (Fig. 6B). Notably, STAT3 silencing remarkably decreased the basal as well as rh-resistin-induced migration and invasion potential of BC cells (Fig. 6A and 6B). Taken together, these findings clearly highlight the role of STAT3 in resistin-induced growth and aggressive phenotypes of BC cells.

## DISCUSSION

Although molecular differences at the genetic level (gene mutations, deletions, etc.) may exist, it is being increasingly appreciated that TME plays an important role in prevalent racial disparity in breast cancer clinical outcome [9]. Data presented herein provide further support to this notion and establish resistin and IL-6 to be important TME-associated factors that could be the key determinant of BC racial disparity. Moreover, the data also delineate molecular association of resistin and IL-6 at the regulatory level and establish STAT3 as a vital mediator in conferring the phenotypic response of resistin in breast tumor cells.

Association of inflammatory cytokines, resistin and IL-6 with breast cancer risk has been reported previously [29-32]. Resistin is shown to positively correlate with increased tumor stage, size and lymph node metastasis in BC [33]. Moreover, an inverse correlation of resistin expression with disease-free as well as overall survival rates has also been reported in BC patients [33]. Similarly, the role of IL-6 has been well studied in several cancer types [27, 30, 34]. IL-6 is secreted by a wide array of immune, endothelial as well as cancer cells in an inducible manner. IL-6-KO mice studies suggested that it played an essential role in the development of immune cells [19]. Significant racially disparate overexpression of IL-6 in BC patients and its association with resistin add another dimension to its pathobiological involvement in breast carcinogenesis. While both resistin and IL-6 exhibited elevated serum levels in AA BC serum as compared to CA patients, we did not detect resistin expression in any of the tested BC cell lines. This might be an effect of long-term culturing of these cells or suggest that the stromal cells are the major source of elevated serum levels of resistin in BC patients. In fact, studies have reported that in human, resistin is generally produced by the recruited immune cells or resident macrophages [12, 13], and its role in differentiation of monocyte to macrophage has also been suggested [13, 35]. Treatment of macrophages with resistin induced the production of the proinflammatory cytokines IL-12 and TNF-α [36]. Moreover, it was also shown that the activation of macrophages with proinflammatory cytokines or endotoxin significantly enhanced the production of resistin [37]. In addition, delivery of endotoxin to human subjects also increased the level of resistin in blood circulation [38]. Thus, the finding of higher resistin levels in BC patients can be explained by the activation of macrophages as a part of the inducing inflammatory process.

Uncontrolled proliferation and aggressiveness are some of the most important characteristics of the cancer cells. Clinically, AA BC patients tend to be diagnosed with more advanced disease and as a result with poorer prognosis as compared to CA BC patients [2, 5]. Our findings demonstrate that stimulation of BC cells with resistin not only enhanced their growth, but also resulted in increased motility and invasive potential of BC cells. These findings are significant and support the notion that elevated expression of resistin in AA BC patients as compared to that in their CA counterpart may underlie the greater aggressiveness of the disease in these patients. Other published studies have also reported a role of resistin in cancer progression, invasion and metastasis [39-43]. Resistin was shown to promote proliferation in prostate cancer [39] and associated with migratory potential and metastasis of chondrosarcoma [42]. Moreover, in a recent study, it was shown that mice receiving anti-resistin antibody had significantly decreased incidence of lung cancer development and metastasis [40]. Furthermore, its role in promoting cell adhesion to the vascular endothelium has also been demonstrated in hepatocellular carcinoma [43]. In some studies, a significance of resistin in the neovascularization process has also been reported. Resistin was shown to stimulate capillary formation [41], and proliferation and migration of endothelial cells [44]. In some earlier studies, an overexpression of resistin receptor, CAP1, has been reported in BC and associated with its pathobiological development [45, 46]. Since we also observed more advanced expression of CAP1 in AA BC cells, it is likely that CAP1 serves as an additional determinant of BC racial disparity. Furthermore, enhanced expression of CAP1 can also explain more potent effect of resistin treatment on IL-6 and STAT3 expression as well as growth and aggressiveness of AA BC cells.

Another significant finding of our study is that we identified oncogenic transcription factor STAT3 to be a crucial mediator of resistin-induced effects on BC cells. STAT3 is a transcription factor, which is constitutively activated in many malignancies, including BC [21]. Role of STAT3 in induction and maintenance of an inflammatory microenvironment during initiation and progression of cancer is well documented [47-49]. It is an influential mediator of tumorigenesis, and has been shown to be crucial for tumor growth, cell proliferation, and apoptosis [50-52]. Moreover, BC cells expressing activated STAT3 exhibit poorer therapeutic response to neo-adjuvant chemotherapy [50]. It is suggested that IL-6 plays an important role in STAT3 activation [23]. In that regard, our finding identifying resistin as a regulator of IL-6 is highly significant. Moreover, we have revealed that resistin, while inducing STAT3 phosphorylation in an IL-6 dependent manner; regulates the expression of STAT3 via some yet unknown mechanism.

In conclusion, we have shown a differential expression of resistin and IL-6 in AA and CA BC patients. Furthermore, our data have revealed a novel mechanistic association of resistin and IL-6 and identified resistin as a novel regulator of STAT3 expression and phosphorylation, where resistin-induced IL-6 expression likely mediates STAT3 activation. In additional novel findings, we have demonstrated STAT3 to be an important mediator of resistin-induced growth and aggressiveness of BC cells. In view of these findings, it appears that resistin and IL-6 may serve as novel, mechanistically-linked set of serum biomarkers exhibiting greater incidence as well as overall levels in AA BC patients compared to that in their CA counterparts. These data also suggest that novel, resistin- and IL-6- targeted therapeutic strategies can be developed to treat aggressive and metastatic breast tumors, and thus reduce the prevalent racial disparity in clinical outcome.

## MATERIALS AND METHODS

### Cell lines and human serum specimens

The human BC cell lines, MCF7, BT-549, MDA-MB-453, MDA-MB-231, MDA-MB-468, and HCC70, were procured from ATCC (Manassas, VA). All the cell lines were maintained in their required Dulbecco's Modified Eagle Medium (DMEM) (GE Healthcare Life Sciences, Logan, Utah), or Minimum Essential Medium (MEM) (GE Healthcare Life Sciences) supplemented with 5 % or 10 % fetal bovine serum (FBS) (Atlanta Biologicals, Lawrenceville, GA), penicillin (100 units/ml) and streptomycin (100 μg/ml) (Invitrogen, Carlsbad, CA) in a humidified atmosphere of 5 % CO_2_ at 37 °C. All the cell lines were tested periodically for mycoplasma and determined to be free from infection. Serum specimens from breast cancer patients of AA and CA racial backgrounds were obtained through Institutional Biobank under the Institutional Review Board-approved protocol.

### Antibodies and siRNAs

Anti–STAT3, pSTAT3-Y705 (rabbit monoclonal) antibodies were purchased from Cell Signaling Technology (Beverly, MA). Antibodies against IL-6, IL-6Rα (rabbit polyclonal), CAP1 (mouse monoclonal) along with anti-mouse and anti-rabbit horseradish peroxidase (HRP)-conjugated secondary antibodies were procured from Santa Cruz Biotechnology (Santa Cruz, CA). β-actin (mouse monoclonal) antibody was purchased from Sigma-Aldrich (St. Louis, MO). Non-targeting scrambled siRNAs (NT-Scr) or STAT3 targeting siRNAs were purchased from GE Dharmacon (Lafayette, CO).

### Treatment and transfection

BC cells (MDA-MB-231 and MDA-MB-468) grown in 96- or 6-well plates were treated with resistin (Phoenix Pharma, Burlingame, CA) or IL-6 (Sigma-Aldrich, St. Louis, MO) as indicated in pertinent figure legends. For transient silencing of STAT3, 60-70 % confluent BC cells were transfected with non-targeting- or STAT3- specific siRNAs (30 nM) using X-treme GENE HP DNA Transfection Reagent (Roche, Indianapolis, IN) as per the manufacturer's instructions. To elucidate the role of IL-6 in resistin-induced STAT3 expression/phosphorylation, BC cells were treated with IL-6 neutralizing antibody (2 μg/ml) (Abcam, Cambridge, MA) or control IgG (2 μg/ml) (Santa Cruz) antibody for 48 h prior to resistin stimulation.

### Enzyme-linked immunosorbent assay (ELISA)

Cytokines profile of pooled serum samples from AA or CA BC patients were analyzed by human cytokine ELISA kit (Signosis, Inc, Santa Clara, CA) as per manufacturer's instruction. Level of resistin and IL-6 in human serum specimens or cultured supernatant of BC cells were analyzed using human resistin and human IL-6 ELISA kits (R&D Systems, MN, USA), respectively, as per manufacturer's instructions.

### Immunoblot analysis

Protein from BC cells was isolated using NP-40 lysis buffer containing protease phosphatase inhibitor. Thereafter, protein samples (20-80 μg) were resolved by SDS-PAGE and subjected to immunoblot analysis as described earlier [53, 54] using proteins specific antibodies. The primary antibodies were used at 1:1000 dilution with the respective HRP labeled secondary antibodies at 1:2500 dilution. β-actin was used at 1:20000 dilution. The signal was detected with ECL plus Western Blotting substrate kit (Thermo Scientific, Logan, UT) using LAS-3000 image analyzer (Fuji Photo Film Co., Tokyo, Japan).

### *In vitro* cell growth assay

BC cells (1×10^4^) were seeded in 96-well plates, and after 24 h cells were transiently transfected with non-targeting or STAT3 targeting siRNAs. 48 h post transfection, cells were treated with resistin (20 ng/ml) and effect on growth was monitored by WST-1 assay (Roche Diagnostics, Mannheim, Germany) as discussed previously [55].

### Plating efficiency assay

For plating efficiency assay, cells (1×10^3^) transfected with NT-Scr or STAT3 targeting siRNA were seeded in 6-well plates and treated with resistin (20 ng/ml). After every third day, medium replaced with fresh culture/treatment media. Following two weeks of culture, colonies were fixed with methanol, stained with crystal violet, photographed and counted using Image analysis software (Gene Tools, Syngene, Frederick, MD).

### Motility and invasion assays

To analyze the effects of the resistin on the migration and invasion ability of STAT3 silenced BC cells, MDA-MB-231 and MDA-MB-468 cells were transfected with NT-Scr or STAT3-targeting siRNAs for 48 h. Post transfection, cells were treated with resistin (20 ng/ml) for next 48 h and seeded (2.5×10^5^/well for migration and 5×10^4^/well for invasion) on top of non-coated (for migration) or Matrigel-coated (for invasion) transwell chamber by following the previously described procedure [28, 56].

### Statistical analysis

All the experiments were performed at least three times, and data are expressed as mean ± SD. Wherever suitable, the data were also subjected to unpaired two tailed Student's t-test and p< 0.05 was considered statistically significant.

## SUPPLEMENTARY MATERIAL, FIGURES, TABLES


