# Supplementary material for: Resistin and interleukin-6 exhibit racially-disparate expression in breast cancer patients, display molecular association and promote growth and aggressiveness of tumor cells through STAT3 activation
Source: Oncotarget. 2015 Mar 14;6(13):11231–41. doi: 10.18632/oncotarget.3591 (PMC4484452; doi:10.18632/oncotarget.3591)
Supplement: Supplementary file 1 [file oncotarget-06-11231-s001.pdf]

Resistin and interleukin-6 exhibit racially-disparate expression in breast cancer patients, display molecular association and promote growth and aggressiveness of tumor cells through STAT3 activation

Supplementary Material

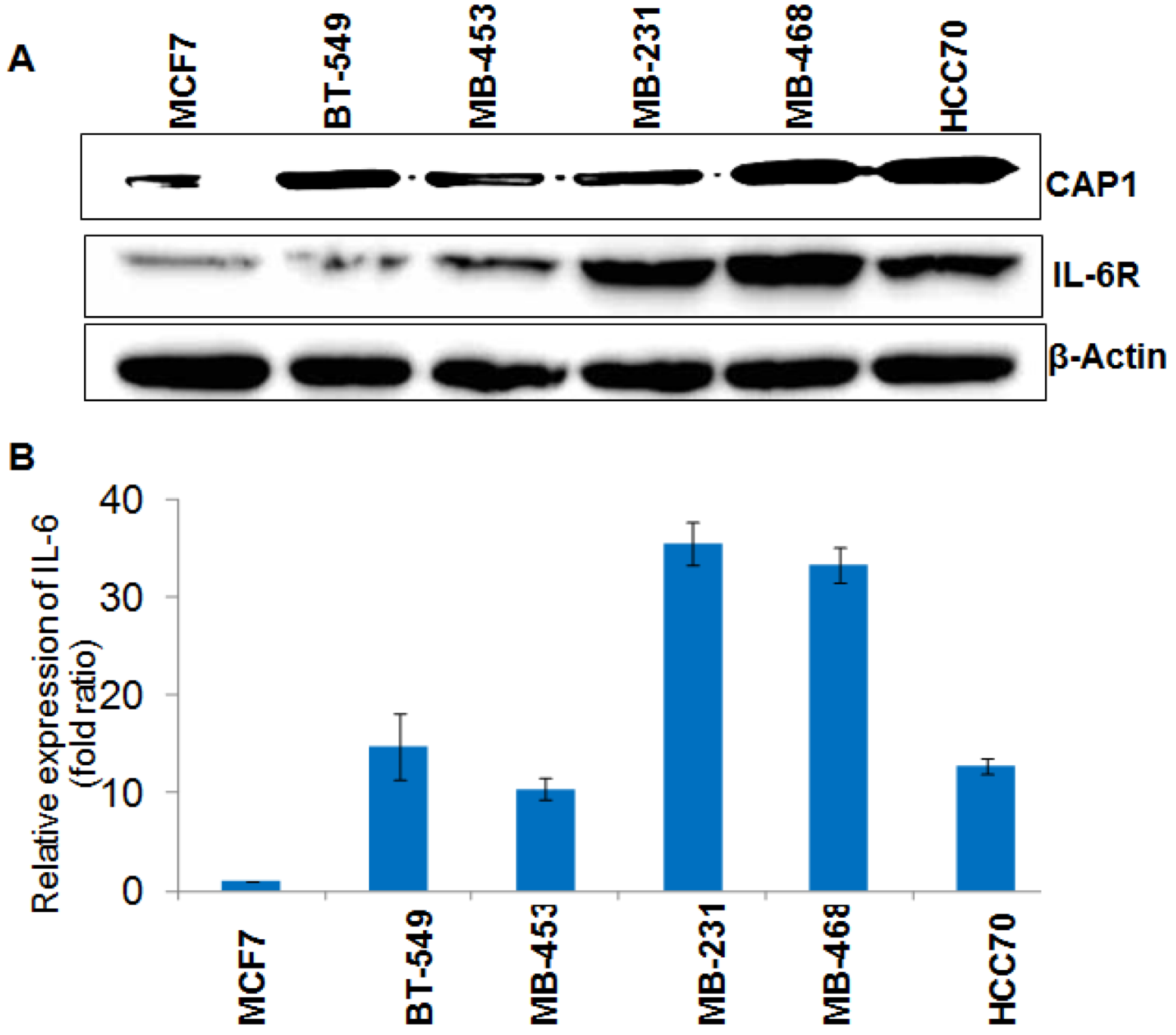

Supplementary Figure 1: Expression of CAP1, IL-6 and IL-6R in BC cell lines. BC cell lines were grown to sub-confluence under regular culture condition, and total protein and culture supernatant was collected. **(A)** immunoblot assay (using 80 µg protein) was performed to examine the expression of CAP1 and IL-6R. β-actin was used as internal control. **(B)** Culture supernatant was subjected to ELISA to measure the level of IL-6.

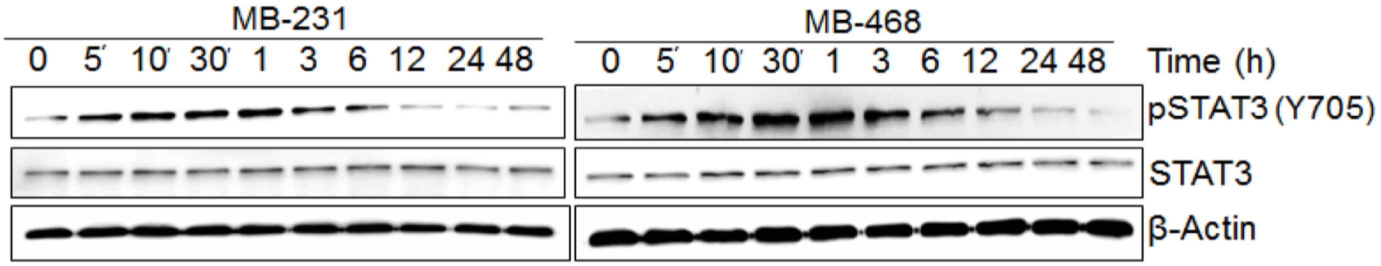

Supplementary Figure 2: IL-6 promotes STAT3 phosphorylation. MDA-MB-231 and MDA-MB-468 BC cells were grown in 6 well plate up to 60-70 % confluence and treated with IL-6 (100 ng/ml) for indicated time intervals. Thereafter, total protein was isolated and subjected to immunoblot to examine the expression of pSTAT3 and STAT3. β-actin was used as internal control.

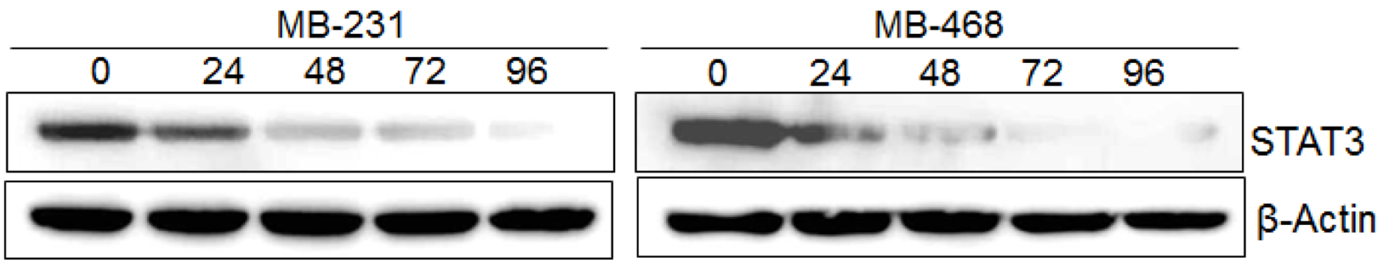

Supplementary Figure 3: Silencing of STAT3 in BC cells. MDA-MB-231 and MDA-MB-468 cells were transfected with STAT3-specific siRNAs, and total protein was extracted at different time intervals (0-96 h). Immunoblot assay was performed to analyze the efficiency and time-course of STAT3 silencing. β-actin was used as internal control.
